# A Case of Progressive Ossifying Fibrodysplasia of Tracheobronchial Respiratory Muscles

**DOI:** 10.1155/2019/5095343

**Published:** 2019-02-11

**Authors:** Nouraly Habib, Anhum Konan, Tra Bi Zamble Olivier Didier

**Affiliations:** ^1^Radiology Department of Mahavoky Atsimo University Hospital, Mahajanga, Madagascar; ^2^Radiology Department of Yopougon University Hospital, 21 BP 632 Abidjan, Côte d'Ivoire; ^3^Radiology Department of Bingerville University Hospital, Abidjan, Côte d'Ivoire

## Abstract

The authors report a case of progressive ossifying myositis (POM) in a 13-year-old boy, revealed by dry cough and dyspnea. Conventional chest x-rays and whole-body CT showed extraskeletal ossification that seems to affect the left bronchial strain and trachea. This lesional topography, if established, not yet described to our knowledge, contrasts with the observations of all the authors, including Munchmeyer, for whom smooth muscles and muscles attached to the skeleton by a single end are spared by the heterotopic ossifications characteristic of the disease. Therefore, this observation raises the question of the ubiquity of muscle ossifications during POM.

## 1. Introduction

Progressive ossifying myositis or fibrodysplasia (POM or POF), also known as Munchmeyer's disease, is an extremely rare genetic disease [1–3]. The heterotopic ossifications of the muscular and connective tissues that characterize it generally spare the smooth muscles and those which do not attach to the skeleton by their 2 ends [3, 4]. We report one case, diagnosed in a teenager with dyspnea and chronic cough, with localization of ossifications in the smooth muscles of the tracheal wall and left bronchial strain.

## 2. Observation

**Figure 1:**
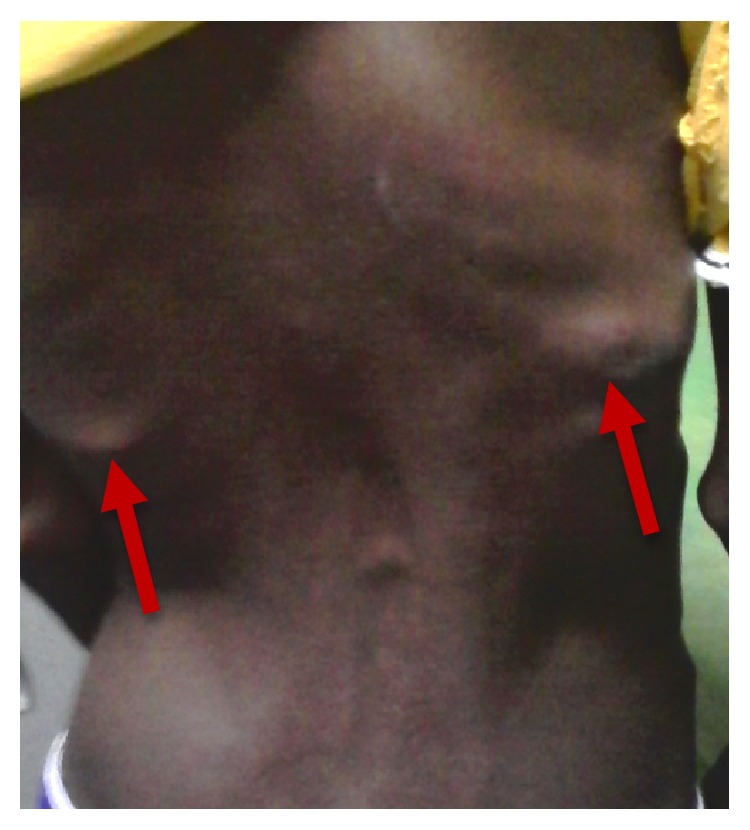
Bilateral back swelling (red arrows) with dextroconvex scoliotic lumbar attitude.

**Figure 2:**
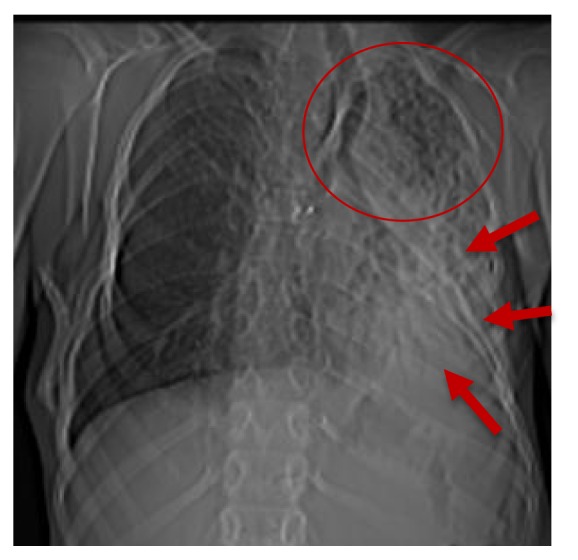
Frontal chest x-ray showing an atelectasis of the left lower lobe (red circle) with alvéolo-interstitial pneumopathy on the homolateral upper lobe (red arrows).

**Figure 3:**
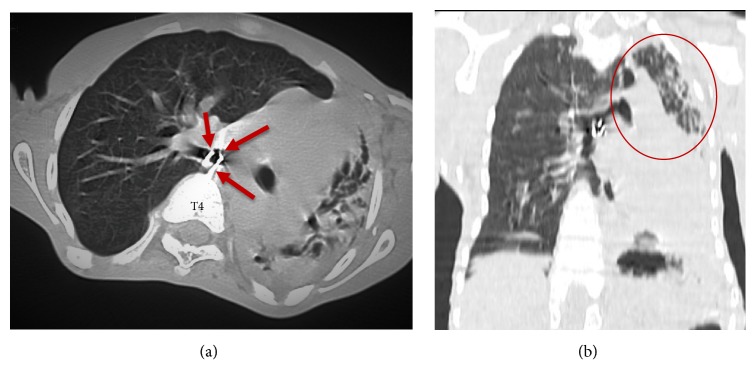
*((a) = axial section and ((b) = coronal reconstruction):* Linear thick extraskeletal, parietal, tracheal, and left bronchial strain calcium formation, circumferential with intraluminal extension, attached to the anterior surface of the T4 vertebral body by a thick extension ((a): red arrows). CT in pulmonary window: atelectasis of the left pulmonary lobes ((b): red circle).

**Figure 4:**
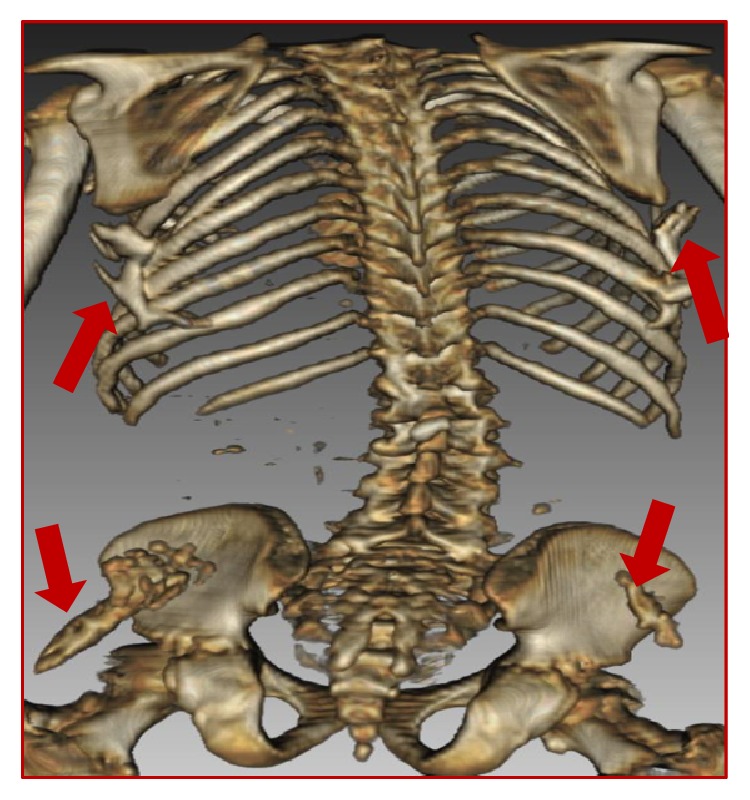
Three-dimensional reconstruction CT: presence of extraskeletal ossifications of the posterior surface of the trunk and the posterior surface of the iliac wings (red arrows).

It is about a boy of 13 years old, cachectic, received for management of a dyspnea occurring on chronic dry cough. Physical examination showed a condensation syndrome of the base of the left pulmonary field. He presented with a fever, physical deformities made of bilateral nonpainful back swelling, dorsolumbar scoliosis (Figure 1), and bilateral hallux valgus. There are also painful joint stiffness in the elbows, shoulders, and hips, which limits passive and active movements. There are no particular medical or family antecedents. The described deformities would have appeared as of the age of 4 years in a child born at term and without malformation, from a pregnancy known as normal. Chest x-ray radiography showed an atelectasis image of the left lower lobe, associated with ipsilateral upper lobar reticular infiltrates with mediastinum attraction on the left side and hyperinflation of the right lung. These abnormalities were accompanied by right extraskeletal laterodorsal bone outgrowth and mediastinal calcification in projection on the tracheal bifurcation (Figure 2). The additional thoracoabdominal CT revealed a thick linear extraskeletal, parietal, tracheal, and left tracheobronchial calcium formation, circumferential with intraluminal extension, attached to the anterior face of the T4 vertebral body by a thick extension. It reduced the calibers of these airways and caused an active collapse of the two left lung lobes downstream. Extraskeletal bone formations were found at dorsal and gluteal soft tissue (Figures 3 and 4). The search for BAAR in sputum was negative. Blood counts, first hour sedimentation rate, C-reactive protein, and serum activity of ASAT, ALAT, GGT, alkaline phosphatases, creatinine, and bilirubinemia were normal.

## 3. Discussion

In total, we noted active atelectasis of the left lung by tracheobronchial stenosis related to ossification of the muscles of the wall of these airways, all occurring in a context of extraskeletal heterotopic ossification of muscular origin, accompanied by characteristic deformities including bilateral hallux valgus. We retained the diagnosis of progressive ossifying myositis. Due to the risk of iatrogenic ossification, no biopsy or other diagnostic or therapeutic surgical procedures were performed. He received nonsteroidal anti-inflammatory drugs, antibiotics associated with respiratory physiotherapy sessions.

Our observation thus reports a case of progressive ossifying myositis with heterotopic ossification that seems to affect the smooth and connective muscle wall of the trachea and the left bronchus strain. This topography of lesions contrasts with Munchmeyer, Hasan, and Kaplan's remarks, according to which progressive ossifying myositis spares smooth muscles and muscles that do not fit on the skeleton by their two extremities such as ocular muscles, diaphragm, tongue, pharynx, and larynx muscles [5–7]. Indeed, in our case, the trachea and left bronchus strain could instead be surrounded by an extrinsic ossification. The pulmonary signs that motivated the consultation in our case, namely cough and dyspnea, are the direct consequence of the luminal obstruction of the airways. Some authors reported pulmonary signs during POM. However, their observations showed pleural irritation related to exuberant ossifications of the chest wall muscles [8, 9]. Ossifications of smooth muscles, especially the respiratory tract, have not yet been mentioned to our knowledge. This is why it would be likely to propose a correlation between this clinico-anatomo-radiological specimen that we describe here and a tracheobronchial POM.

## 4. Conclusion

This case of progressive ossifying fibrodysplasia with a potential ossification of the tracheobronchial airway wall muscles leads us to question the ubiquitous nature of heterotopic ossifications during POM.
